# Programmed Cell Death Protein 1 Contributes to Oral Cancer Pain *via* Regulating Tumor Necrosis Factor Alpha in the Spinal Trigeminal Nucleus Caudalis

**DOI:** 10.2174/1570159X23666241209160039

**Published:** 2024-12-09

**Authors:** Runyi Mao, Sufang Liu, John C. Dolan, Brian L. Schmidt, Feng Tao

**Affiliations:** 1 Department of Biomedical Sciences, Texas A&M University School of Dentistry, Dallas, Texas, USA;; 2 NYU Dentistry Translational Research Center, New York University College of Dentistry, New York, NY, USA;; 3 Pain Research Center, New York University, New York, NY, USA

**Keywords:** Oral cancer pain, functional allodynia, hypersensitivity, programmed cell death protein 1, spinal trigeminal nucleus caudalis, tumor necrosis factor-alpha

## Abstract

**Background:**

Oral cancer causes intense pain at the primary site, and such pain can impair oral functions. However, the underlying mechanisms for oral cancer pain are still not fully understood. In the present study, it is investigated whether programmed cell death protein 1 (PD-1) is involved in the development of oral cancer pain.

**Methods:**

RMP1-14, a specific anti-PD-1 antibody, was injected into spinal trigeminal nucleus caudalis (Sp5C) and measured pain behaviors using von Frey filaments and dolognawmeter. Western blotting and immunofluorescence staining were performed to analyze the expression of PD-1 and tumor necrosis factor alpha (TNFα) in the Sp5C.

**Results:**

It was observed that the PD-1 antibody significantly inhibited mechanical hypersensitivity and functional allodynia in our oral cancer pain mouse model. Moreover, we found that TNFα was highly upregulated in the Sp5C following the induction of oral cancer pain and that intra-Sp5C injection of the PD-1 antibody diminished the upregulation of TNFα. It was found that genetic deletion of TNFα or its receptor antagonism synergized the analgesic effect of PD-1 antibody on oral cancer pain.

**Conclusion:**

Our results suggest that PD-1 in the Sp5C contributes to oral cancer pain by altering TNFα signaling in the trigeminal nociceptive system, and PD-1 could be targeted to develop a novel approach for oral cancer pain management.

## 1 INTRODUCTION

Pain is the most common complaint in patients with oral squamous cell carcinoma (SCC) [1-3]. As an early symptom, oral cancer pain at the primary site is closely related to tumor development in the region of the tongue and floor of the mouth. Hence, pain symptoms can be a critical indicator of cancer progression and prognosis of cancer treatment [4]. Oral cancer pain is triggered not only by the physical occupancy of the tumor but also by several mediators released from carcinoma cells and the microenvironment [5]. Accumulating evidence has shown that tissue injury and inflammation can increase pain sensitization by releasing inflammatory cytokines, such as tumor necrosis factor-alpha (TNFα) [6-9]. Cancer-relevant immune cells secrete several neuro-immune mediators and then regulate peripheral nociceptors, thereby inducing hyperalgesia and allodynia [10, 11]. It has been reported that TNFα is involved in the development of cancer pain in an oral cancer pain model [11].

Programmed cell death protein 1 (PD-1) and its ligand (PD-L1) have been discovered as immune checkpoints for cancer immunotherapy [12]. Accordingly, the PD-L1/PD-1 immune checkpoint has become one of the most revolutionary findings for developing new treatments for different cancers, including melanoma and cancers in the lung, head & neck, kidney, and bladder [13]. Moreover, activation of PD-1 signaling in the nervous system enhances pain by suppressing T-cell function in melanoma [14]. This signaling also regulates the polarization of macrophages/microglia [15]. Interestingly, TNFα blockade can overcome the resistance to anti-PD-1 therapy in experimental melanoma [16]. Furthermore, it is proposed that TNFα may serve as an immune checkpoint in cancer development [17]. However, it is unknown whether PD-1 plays a role in the pathogenesis of oral cancer pain and, if so, how the PD-1 signaling contributes to such pain.

In the present study, we reveal the role of trigeminal PD-1 in oral cancer pain by examining the effect of specific PD-1 antibodies and investigating the underlying molecular mechanism.

## 2 MATERIALS AND METHODS

### 2.1 Animals

In this study, 8-10 weeks male C57BL/6 wild-type (WT) and TNFα knockout (KO) mice purchased from the Jackson Laboratory were used. The mice were housed under standard conditions with a 12-hour light-dark cycle, with water and food pellets available as *ad libitum*. Mouse acclimation was conducted for a minimum of one week before behavioral experiments and the mice received additional acclimation for 30-60 min before each behavioral test. All animal experiments were approved by the Institutional Animal Care and Animal Use Protocol: 2021-0086 Use Committee at Texas A&M University School of Dentistry and all efforts were made to minimize pain or discomfort and to reduce the number of animals used. Our experiments were performed in accordance with the National Institutes of Health guide for the care and use of laboratory animals.

### 2.2 Oral Cancer Pain Model

We prepared our oral cancer pain model as described previously [18] with minor modifications. In brief, dysplastic DOK from ATCC, #PCS-200-014 oral keratinocytes (DOK, a control cell line) and HSC-3 from Sigma, #SCC-193, Human Squamous Carcinoma (HSC-3) cells were cultured in 75 mm^2^ flasks at 37°C with 5% CO_2_ in Dulbecco Modified Eagle medium (Gibco, Waltham, MA) supplemented with 10% fetal bovine serum and penicillin/streptomycin (50 U/ml). After cells reached 70-80% confluency, the culture medium was changed to a serum-free medium. The supernatant of the culture medium was collected following 72-h incubation. Next, the supernatant (50 µl) was injected into one side of the mouse tongue, and the needle remained in place for an additional 15 s to avoid leakage of the supernatant.

### 2.3 Intra-Sp5C Microinjection

We performed microinjection of RMP1-14 (Bio X Cell) and/or R-7050 (Cayman Chemical) into Sp5C of mice. In brief, mice were anesthetized with 2% isoflurane and placed onto a stereotaxic instrument. After their head skin was cut and appropriate hemostasis achieved using a sterile technique, a hole in the skull was drilled, and 0.5 μl of RMP1-14 (3.5 μg) and/or 0.5 μl of R-7050 (0.1 mM in 0.9% saline) were injected into Sp5C according to predetermined coordinates (AP, -8.0 mm; ML, 1.5 mm; DV, 4.5 mm) [19]. The intra-Sp5C microinjection was done within 2 min, and the needle remained in place for an additional 1 min. At the end of the experiments, the microinjection site was confirmed histologically.

### 2.4 Mechanical Hypersensitivity Test

The calibrated von Frey filaments were used as described in our previous studies [20-28] to test orofacial mechanical hypersensitivity before and after intra-tongue injection of cell culture medium supernatants or intra-Sp5C injection of RMP1-14 and/or R-7050. The mice were placed into a 10-cm-long restraining Plexiglas cylinder that prevented them from turning around but allowed them to poke their heads and forepaws. After acclimation for 10 min, the filament was applied to skin areas innervated by the trigeminal nerve V3 branch. Each filament was applied five times to the V3-innervated skin area for 1-2 s with a 10s interval, starting from the lowest force of filament (0.08 g) and continuing in ascending order. A positive‭ ‬response to the stimulus was defined as a sharp withdrawal of the head. The head withdrawal threshold was then calculated as the force at which the positive response occurred in three of five stimuli.

### 2.5 Functional Allodynia Test

Dolognawmeters were used to measure functional allodynia, as described in our previous studies [20, 26, 29]. Briefly, each mouse was placed into a tube with a series of two obstructing dowels. The mice were allowed to gnaw completely through both dowels to escape the device. Each dowel was connected to a digital timer that recorded when the mouse severed each dowel in series. The duration of time required to sever both dowels was operationally defined as the gnaw time.

### 2.6 Western Blotting

We harvested mouse Sp5C tissues under isoflurane anesthesia at 45 min following different treatments. The expression of PD-1 or TNFα was analyzed with a quantitative Western blot analysis, and the affinity-purified antibodies against PD-1 (1:1000, rabbit; Sigma, #PRS4065) and TNFα (1:2000, ThermoFisher, #701135) were used. β-actin served as a loading control in all Western blot experiments. The intensities of bands in the Western blotting were quantified with densitometry.

### 2.7 Immunofluorescence Staining

Following the perfusion, Sp5C-containing brain sections were cut at 20 µm with a cryostat (CM1950, Leica, Chicago, IL). Free-floating slices were blocked in a 5% normal goat serum for 1 h, followed by incubation with primary antibodies overnight at 4°C. Next, the slices were washed and placed in a corresponding secondary antibody conjugated to Alexa Fluor 488 or Cy3 for 1 h at room temperature. The following primary antibodies were used in this study: anti-PD-1 antibody (1:200, rabbit; Sigma, #PRS4065), anti-neuron-specific nuclear protein (NeuN, 1:400, Cell Signaling, 12943S), anti-ionized calcium-binding adapter molecule 1 (Iba-1, 1:400, FUJIFILM Wako Chemicals, #019-19741), and anti-glial fibrillary acidic protein (GFAP, 1:800, EMD Millipore, #AB5541). Immunofluorescent imaging was observed under a Leica fluorescence microscope (DMi8, Leica), and the image analysis was performed using ImageJ software.

### 2.8 Statistical Analysis

Data are expressed as means ± SEM. Western blotting data were analyzed with a student’s t-test. One-way and two-way ANOVA with repeated measures followed by appropriate post-hoc tests were performed for behavioral testing data. *P* < 0.05 was considered statistically significant.

## 3 RESULTS

### 3.1 PD-1 is Expressed in Neurons of Mouse Sp5C

**Fig. (1):**
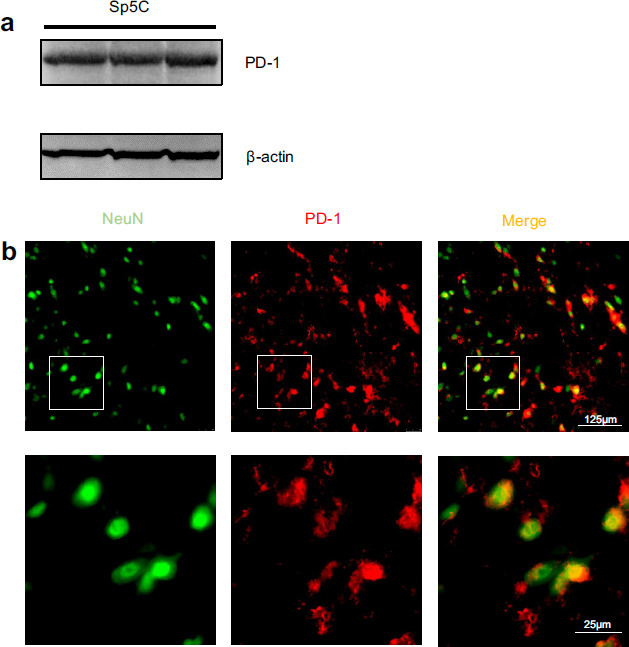
PD-1 is expressed in neurons of mouse Sp5C. (**a**) Western blot analysis showed that PD-1 was strongly expressed in the mouse Sp5C tissues (n = 3). (**b**) Double immunofluorescence staining further showed that most PD-1-positive cells in the Sp5C were co-labeled with NeuN (a neuronal marker). The fluorescence images of the lower panel display the respective boxed area of the upper panel at higher magnification. The immunofluorescence staining experiment was repeated three times to confirm the data shown in the figure. Scale bars, 125 µm for lower magnification in the upper panel and 25 µm for higher magnification in the lower panel.

To examine the expression of PD-1 in the Sp5C, Western blotting, and double immunofluorescence staining were performed. Our Western blotting data showed that PD-1 was strongly expressed in the mouse Sp5C tissues (Fig. **1a**). The double immunofluorescence staining further revealed that PD-1 in the Sp5C was primarily expressed in neurons (Fig. **1b**). Using antibodies against glial markers, we further observed that Sp5C PD-1 was not expressed in GFAP-labeled astrocytes, but some PD-1-positive cells were co-expressed with the microglial marker Iba-1 (Fig. **S1**). The percentage of PD-1-positive cells colocalized with the neuronal marker NeuN and the microglial marker Iba-1 in the Sp5C was 65.38% and 31.90%, respectively.

### 3.2 Intra-Sp5C Injection of PD-1 Antibody Inhibits Oral Cancer Pain

**Fig. (2):**
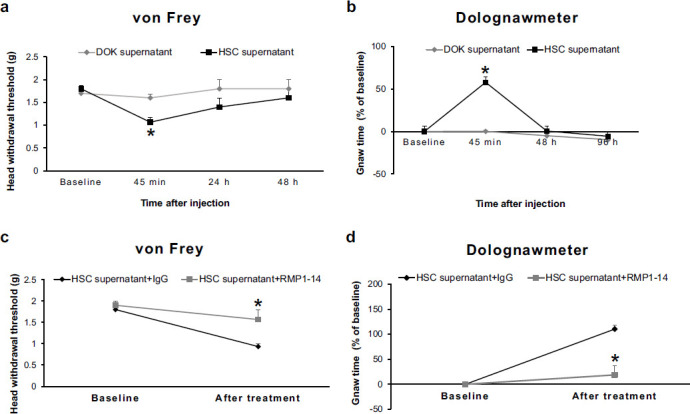
Intra-Sp5C injection of PD-1 antibody inhibits oral cancer pain. (**a**) In the von Frey test, intra-tongue injection of the medium supernatant of HSC-3 cell culture significantly decreased head withdrawal thresholds at 45 min post-injection compared to those in the DOK supernatant-treated control group, and the decreased head withdrawal thresholds returned to the baseline level at 48 h post-injection (n = 12). *P < 0.05 *vs.* the DOK supernatant-treated control group. (**b**) In the dolognawmeter test, gnaw time in the HSC supernatant-treated group significantly increased at 45 min post-injection compared to that in the DOK supernatant-treated control group, and the increased gnaw time returned to the baseline level at 48 h post-injection (n = 6). *P < 0.05 *vs.* the DOK supernatant-treated control group. (**c** and **d**) Intra-Sp5C injection of the PD-1 antibody blocked the development of the HSC supernatant-induced oral cancer pain compared to the IgG-treated control group in both von Frey test (**c**) and dolognawmeter test (**d**) (n = 6). *P < 0.05 *vs.* the IgG-treated control group.

To determine the role of PD-1 in oral cancer pain, we prepared a mouse oral cancer pain model and examined the effect of intra-Sp5C injection of the PD-1 antibody RMP1-14 on such pain. We measured mechanical hypersensitivity and functional allodynia using von Frey and dolognawmeter tests, respectively. In the von Frey test, we observed that intra-tongue injection of the medium supernatant of HSC-3 cell culture significantly decreased head withdrawal thresholds at 45 min post-injection compared to those in the control group with an intra-tongue injection of the medium supernatant of DOK cell culture (Fig. **2a**) and the decreased head withdrawal thresholds returned to the baseline level at 48 h post-injection (Fig. **2a**). In the dolognawmeter test. It was observed that the gnaw time in the HSC-3 supernatant-treated group significantly increased at 45 min post-injection compared to that in the DOK supernatant-treated control group (Fig. **2b**) and the increased gnaw time returned to the baseline level at 48 h post-injection (Fig. **2b**).

Next, the PD-1 antibody RMP1-14 or IgG control was simultaneously injected into unilateral Sp5C of the mice while oral cancer pain was induced with HSC-3 supernatant. We found that intra-Sp5C injection of the PD-1 antibody blocked the development of the HSC-3 supernatant-induced oral cancer pain compared to the IgG-treated control group in both von Frey and dolognawmeter tests (Fig. **2c** and **d**).

### 3.3 Intra-Sp5C Injection of PD-1 Antibody Diminishes the Upregulation of TNFα During Oral Cancer Pain

**Fig. (3):**
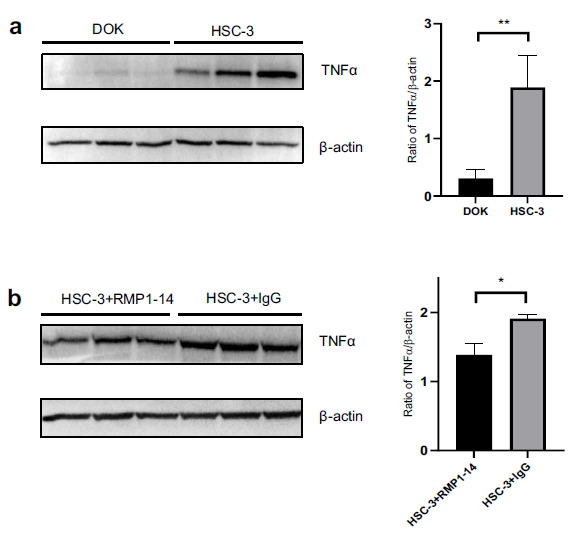
Intra-Sp5C injection of PD-1 antibody diminishes the upregulation of TNFα during oral cancer pain. (**a**) Our Western blotting data showed that intra-tongue injection of HSC-3 supernatant robustly enhanced the expression of TNFα in the Sp5C at 45 min post-injection compared to that in the DOK supernatant-treated control group (n = 3). **P < 0.01 *vs.* the DOK supernatant-treated control group. (**b**) The simultaneous treatment with the PD-1 antibody RMP1-14 significantly reduced the HSC-3 supernatant-increased Sp5C TNFα level during oral cancer pain compared to the IgG-treated control group (n = 3). *P < 0.05 *vs* the IgG-treated control group.

To explore whether TNFα in the Sp5C is involved in oral cancer pain and mediates the effect of the PD-1 antibody treatment on such pain, we harvested Sp5C tissues at 45 min following the treatment and carried out Western blot analysis. Our Western blotting data showed that intra-tongue injection of HSC supernatant robustly enhanced the expression of TNFα in the Sp5C at 45 min post-injection compared to that in the DOK supernatant-treated control group (Fig. **3a**). More importantly, it was found that simultaneous treatment with the PD-1 antibody RMP1-14 significantly reduced the HSC supernatant-increased Sp5C TNFα level during oral cancer pain compared to the IgG-treated control group (Fig. **3b**).

### 3.4 Genetic Deletion of TNFα or its Receptor Antagonism Synergizes the Analgesic Effect of PD-1 Antibody on Oral Cancer Pain

**Fig. (4):**
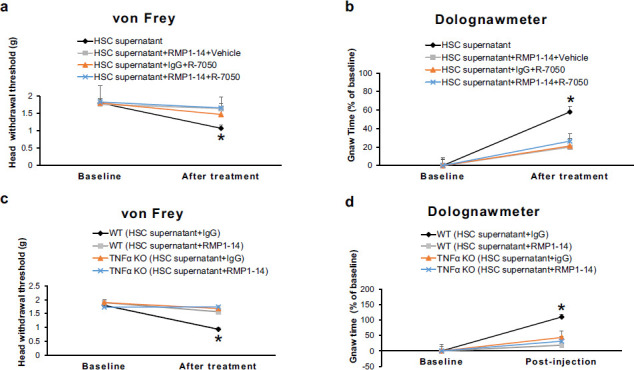
Genetic deletion of TNFα or its receptor antagonism synergizes the analgesic effect of PD-1 antibody on oral cancer pain. **A** and **b** Intra-Sp5C injection of R-7050, a cell-permeable TNFα receptor antagonist, inhibited the HSC supernatant-induced oral cancer pain in both von Frey test (**a**) and dolognawmeter test (**b**) (n = 6-7). *P < 0.05 *vs.* the “HSC supernatant+RMP1-14+Vehicle” group, “HSC supernatant+ IgG+R-7050” group, or “HSC supernatant+RMP1-14+R-7050” group. **c** and **d** Genetic deletion of TNFα inhibited the HSC supernatant-induced oral cancer pain in both von Frey test (**c**) and dolognawmeter test (**d**). (n = 5-6). *P < 0.05 *vs.* the “WT (HSC supernatant+RMP1-14)” group, “TNFα KO (HSC supernatant+IgG)” group, or “TNFα KO (HSC supernatant+RMP1-14)” group.

To determine the role of TNFα signaling in the analgesic effect of the PD-1 antibody RMP1-14 on oral cancer pain, we used selective TNFα receptor antagonism or employed TNFα KO mice. It was observed that either intra-Sp5C injection of R-7050, a cell-permeable TNFα receptor antagonist, or genetic deletion of TNFα inhibited the HSC supernatant-induced oral cancer pain in both von Frey and dolognawmeter tests (Figs. **4a**-**d**), and that combining TNFα signaling interruption with RMP1-14 treatment synergized their effects on oral cancer pain (Figs. **4a**-**d**).

## 4 DISCUSSION

In the present study, it was demonstrated that neuronal PD-1 is involved in the pathogenesis of oral cancer pain. Our results show that PD-1 is mostly expressed in Sp5C neurons, and intra-Sp5C injection of a specific PD-1 antibody can inhibit oral cancer pain. We further reveal that the PD-1 antibody treatment can diminish the upregulation of TNFα in the Sp5C during oral cancer pain. Moreover, genetic deletion of TNFα or its receptor antagonism synergizes the analgesic effect of PD-1 antibody on oral cancer pain. These results suggest that the neuronal PD-1 contributes to such pain by regulating TNFα signaling in the Sp5C. Therefore, targeting Sp5C PD-1 could be used to develop a novel therapy for oral cancer pain.

It has been reported that both PD-1 and PD-L1 are expressed in the nervous system and that PD-L1 plays an important role in pain modulation *via* PD-1 [14]. However, it is unknown whether TNFα mediates the role of PD-L1/PD-1 signaling in oral cancer pain. Previous studies have shown that TNFα is involved in different types of pain [30, 31]. Moreover, TNFα has served as an immune checkpoint in immunotherapy. Specifically, an armed adenovirus can be used in anti-PD-1 immune therapy [32]. Additionally, a TNFα blockade has been proven as a combined treatment for overcoming PD-1 antibody resistance [16]. The accumulating evidence strongly implies that TNFα has a close relationship with the PD-1 signaling pathway. In our study, we provide additional evidence to show that TNFα in the Sp5C could mediate the analgesic effect of PD-1 antibody treatment on oral cancer pain.

PD-L1/PD-1 signaling can interact with several other inflammatory cytokines (such as IL-17) to regulate pain [33-35]. Furthermore, previous studies have indicated that the immune system and nervous system are connected *via* glial cells, which play a critical role in pain modulation [6, 9]. Interestingly, glial cells can regulate immune function through PD-L1 [8]. Therefore, the interactions among neurons, glia, and immune cells, as well as the involvement of different inflammatory cytokines, need to be further studied in terms of their roles in PD-L1/PD-1 signaling produced regulation of oral cancer pain. These future investigations will help us understand the mechanisms underlying neural-immune interactions in the development and maintenance of oral cancer pain.

## CONCLUSION

In conclusion, our current study suggests that the PD-1 signaling pathway in the central nervous system may be targeted to develop a novel approach for pain management in patients with oral cancer. This approach could be applied to other types of cancer pain as well.

## AUTHORS' CONTRIBUTIONS

All authors read and approved the manuscript. R.M. and F.T.: conceptualization; R.M., S.L., J.C.D., B.L.S., and F.T.: methodology and data analysis; R.M.: writing (original draft); F.T. and S.L.: writing (editing).

## Data Availability

All data generated during this study are included in this published article.

## LIST OF ABBREVIATIONS

SCC: Squamous Cell Carcinoma
TNFα: Tumor Necrosis Factor-alpha
WT: Wild-type
