# Supplementary material for: Programmed Cell Death Protein 1 Contributes to Oral Cancer Pain via Regulating Tumor Necrosis Factor Alpha in the Spinal Trigeminal Nucleus Caudalis
Source: Curr Neuropharmacol. 2024 Dec 9;23(5):594–601. doi: 10.2174/1570159X23666241209160039 (PMC12163463; doi:10.2174/1570159X23666241209160039)
Supplement: Supplementary file 1 [file CN-23-5-594_SD1.pdf]

## Supplementary Material

# Programmed Cell Death Protein 1 Contributes to Oral Cancer Pain *via* Regulating Tumor Necrosis Factor Alpha in the Spinal Trigeminal Nucleus Caudalis

Runyi Mao[1], Sufang Liu[1,*], John C. Dolan[2], Brian L. Schmidt[2,3] and Feng Tao[1,*]

*[1]Department of Biomedical Sciences, Texas A&M University School of Dentistry, Dallas, Texas, USA; [2]NYU Dentistry Translational Research Center, New York University College of Dentistry, New York, NY, USA; [3]Pain Research Center, New York University, New York, NY, USA*

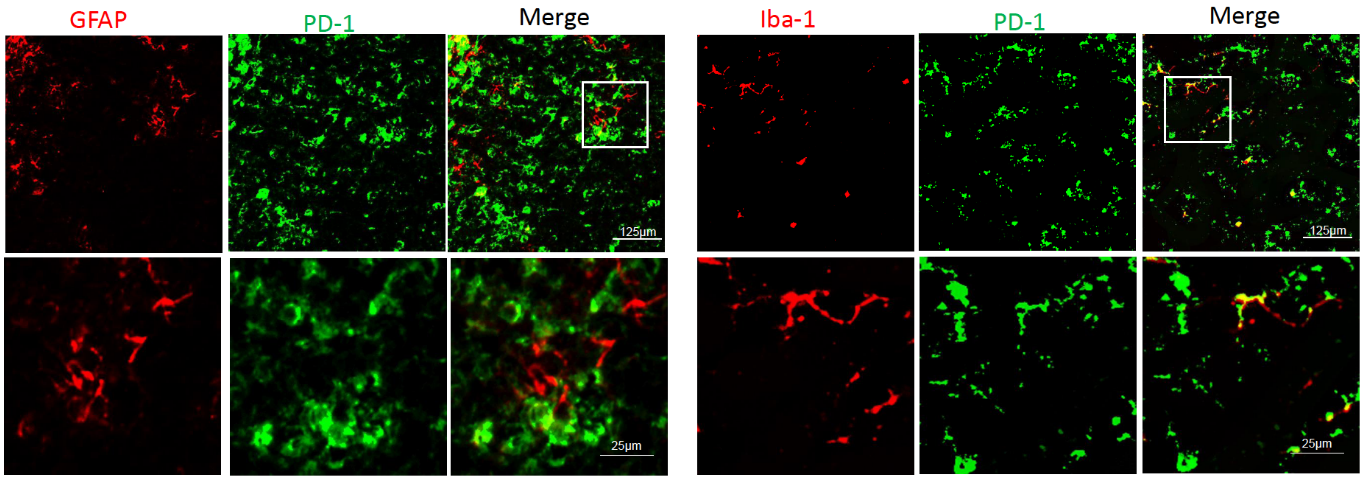


**Fig. (S1).** PD-1 is expressed in some glia but not astrocytes of mouse Sp5C. Double immunofluorescence staining showed that PD-1-positive cells in the Sp5C were not co-labeled with GFAP (an astrocyte marker), and some Sp5C PD-1-positive cells were co-labeled with Iba-1 (a microglial marker). The fluorescence images of the lower panel display the respective boxed area of the upper panel at higher magnification. The immunofluorescence staining experiment was repeated three times to confirm the data shown in the figure. Scale bars, 125 μm for lower magnification in the upper panel and 25 μm for higher magnification in the lower panel.

1875-6190/25 $65.00+.00 © 2025 Bentham Science Publishers

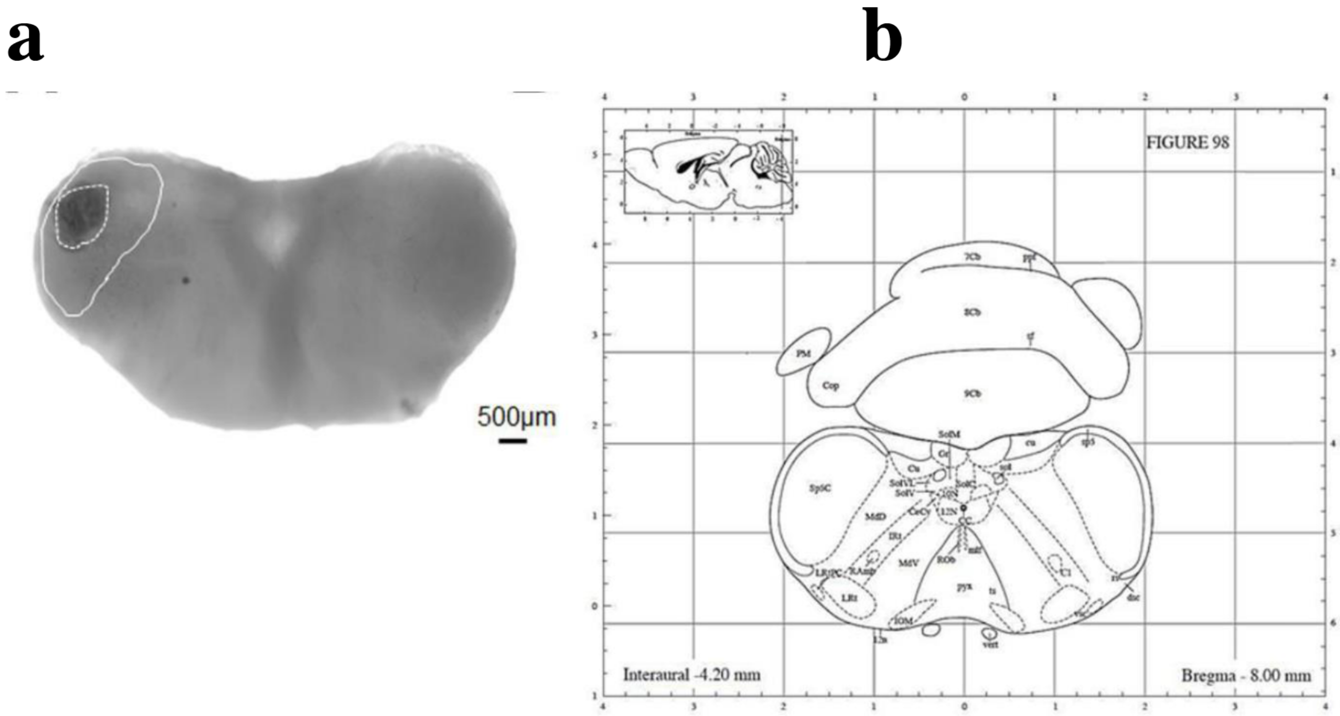


**Fig. (S2).** Localization of microinjection within the Sp5C. **a** 1 µl of methylene blue (0.05%, w/v) was injected into one side of Sp5C. We observed that the dye was displayed in the ipsilateral Sp5C, and the average diameter of the diffusing area (circled area in the Sp5C) for the microinjection is 1.23 ± 0.11 mm within the ipsilateral Sp5C. **b** The coronal section showing Sp5C in the mouse brain atlas at Bregma -8.00 mm. The slide with dye diffusion in (**a**) displays a similar section to the one in (**b**).
